# Artificial intelligence for full-cycle clinical management in orthokeratology: a comprehensive review

**DOI:** 10.3389/fmed.2026.1950432

**Published:** 2026-10-12

**Authors:** Xiaotong Wang, Haojiang Yang

**Affiliations:** 1 School of Medicine, Shenzhen University, Shenzhen, China; 2 Department of Ophthalmology, Shenzhen Second People's Hospital, Shenzhen, China

**Keywords:** artificial intelligence, deep learning, myopia control, orthokeratology, precision medicine

## Abstract

The global myopia epidemic, especially among children, poses a major public health burden. Orthokeratology (OK) is an established intervention for slowing myopia progression, yet its conventional practice is constrained by variable treatment responses, an experience-dependent fitting process, and the need for vigilant long-term safety monitoring. Artificial intelligence (AI) offers an approach to enhance precision, efficiency, and accessibility across the entire OK care cycle. This review assesses AI applications in OK, encompassing pre-treatment efficacy prediction, AI-assisted lens fitting and automated fit assessment during treatment, and post-treatment safety monitoring with complication risk alert. Evidence indicates that AI models perform comparably to, and in some tasks surpass, human experts in predicting outcomes and determining optimal lens parameters. However, translation from research to routine practice is hindered by limited data quality and diversity, the opacity of complex algorithms, insufficient external validation, and unresolved ethical concerns. Progress depends on building large-scale, diverse datasets, developing interpretable and robust models, and fostering effective human-AI collaboration.

## 1 Introduction

### 1.1 Global myopia epidemic and public health burden

Myopia is no longer merely a refractive error but a chronic ocular disease that requires long-term management due to its progressive nature and sight-threatening complications. Projections by Holden et al. suggest that by 2050, nearly half of the global population (4.758 billion) could be myopic, with about 938 million having high myopia (1). This demographic shift implies that healthcare systems must prepare for sustained, lifelong demand for ophthalmic care from a growing and aging myopic population.

The most severe consequences of myopia arise from high myopia, which significantly increases the risk of sight-threatening complications such as myopic maculopathy, a leading cause of irreversible blindness (2). The problem is most urgent in East and Southeast Asia, where 80%–90% of teenagers are myopic and 10%–20% already have high myopia (2, 3). The rapid increase in myopia prevalence in these regions, driven largely by environmental and lifestyle factors such as intensive education and reduced outdoor time, underscores the need for effective interventions that address both risk identification and disease modification (4). As this cohort ages, an estimated one in three individuals with high myopia is expected to develop visual impairment, heralding a substantial wave of disability (3).

Despite this urgency, most clinical trials for myopia control have focused on low-to-moderate cases, leaving a critical evidence gap for managing high myopia (3). This gap reinforces the importance of early intervention in childhood, as children with early onset exhibit the fastest progression and the highest lifetime risk of severe complications (1, 3).

### 1.2 Efficacy and role of orthokeratology in myopia management

Orthokeratology (OK) is an established intervention for slowing myopia progression in children. Systematic reviews confirm its efficacy in reducing axial elongation compared to single-vision spectacles (5, 6). When evaluated against other control strategies through network meta-analysis, OK demonstrates superior efficacy relative to alternatives such as low-dose atropine and multifocal soft contact lenses (7).

Recent mechanistic insights have further elucidated the biological pathways underlying OK's efficacy. Han et al. systematically reviewed the evidence base for OK's myopia control mechanisms and proposed a “three-tier regulatory network” model encompassing optical defocus, corneal biomechanical stimulation, retinal-choroidal neurovascular coupling, and scleral remodeling (8). The review also highlighted the emerging role of AI in integrating multimodal data to resolve individual response heterogeneity and optimize clinical decision-making.

However, this efficacy is accompanied by important limitations. While effective, OK therapy requires strict hygiene and regular monitoring to mitigate risks of serious complications like microbial keratitis (9). Treatment response also exhibits substantial interindividual variability, with factors including baseline age and corneal characteristics significantly influencing myopia control outcomes (10).

Thakur et al. reported that orthokeratology combined with atropine reduces axial elongation by an additional 0.09–0.29 mm compared with orthokeratology alone over 12–24 months, yet the mechanisms underlying this additive effect remain unclear and long-term safety data are scarce (11).

These challenges in safety management and efficacy predictability underscore the clinical need for improved approaches to optimize patient selection and risk mitigation in OK practice.

### 1.3 Challenges in conventional OK lens clinical practice

A primary obstacle lies in the subjective and time-consuming nature of the fitting process. As highlighted in the BCLA CLEAR report by Vincent et al., conventional fitting relies heavily on iterative trial-and-error with diagnostic lenses, a process inherently dependent on practitioner experience and skill (12). This experiential dependency introduces variability, where inappropriate parameter selection can compromise visual outcomes, patient comfort, and safety. The process demands considerable time and resources, limiting patient throughput and accessibility.

Even after a successful fit, the risk of complications necessitates vigilant long-term monitoring. Maseedupally et al. directly linked corneal toricity to treatment zone decentration, a common issue that can induce optical aberrations and compromise visual quality (13). More critically, Cope et al. documented the devastating consequences of Acanthamoeba keratitis among rigid gas permeable lens wearers, a risk intimately tied to hygiene practices and water exposure (14). These studies collectively underscore that the safety profile of OK is not guaranteed but actively managed through patient compliance and consistent clinical supervision, both of which are variable and difficult to control.

Furthermore, the well-documented heterogeneity in treatment response among children translates directly into a clinical prediction challenge (10). The inability to reliably identify poor responders before initiating therapy leads to inefficiencies and potential disappointment. Clinicians currently lack tools for truly personalized, predictive decisions, often resulting in reactive rather than proactive management.

In summary, conventional OK practice is constrained by an experience-dependent fitting process, persistent safety concerns, and unpredictable individual responses. These limitations create a clear impetus for data-driven, objective tools to augment clinical decision-making and standardize care.

### 1.4 The promise of AI in ophthalmology

Artificial intelligence has demonstrated substantive progress in specific ophthalmic domains, yet its successes are contextual and bounded by the limitations of the underlying studies. In diabetic retinopathy (DR) screening, Kirkizlar et al. conducted a prospective multi-center study evaluating an automated system against a standardized reference, reporting 97.5% sensitivity and 98.5% specificity for detecting referable DR (15). This study provided early, robust evidence that AI could automate a well-defined screening task. However, its performance was intrinsically linked to the quality of fundus photographs and the diagnostic criteria of the specific telemedicine network in which it was validated.

Building on this, Salongcay et al. performed an independent external validation of a deep learning algorithm, confirming its diagnostic accuracy comparable to retinal specialists, with an area under the curve (AUC) of 0.954 (16). This work underscored the critical importance of external validation in assessing real-world generalizability. A key limitation noted was the algorithm's performance dependency on image gradability, a vulnerability when faced with poor-quality data.

Beyond still image analysis, Mueller et al. investigated a remote eye disease assessment platform, a precursor to integrated AI diagnostics (17). While their system demonstrated the logistical feasibility of teleophthalmology, its diagnostic accuracy for conditions beyond DR, such as glaucoma, was limited. This study illustrated that while AI can enable new care pathways, its diagnostic scope remains constrained by the specific diseases and data modalities on which it is trained.

Finally, Qian et al. organized a competitive challenge for diagnosing myopic maculopathy, pushing AI into the domain of complex pathologic sequelae of high myopia (18). The winning algorithm demonstrated that machine perception could identify subtle, sight-threatening lesions, attaining a top AUC of 0.972. However, competition performance does not guarantee seamless clinical workflow integration or reliable real-world screening performance.

Beyond posterior segment diseases, AI has also demonstrated promising performance in diagnosing anterior segment and ocular surface conditions, including dry eye, keratitis, and corneal infections (19). Advances in AI-assisted diagnosis of ocular surface diseases have been systematically reviewed, covering applications ranging from dry eye disease to infectious keratitis (20). In the domain of refractive surgery, AI applications have also gained substantial momentum, with machine learning models being developed for preoperative planning, intraoperative guidance, and postoperative outcome prediction (20).

More broadly, the application of AI to predict refractive error onset and progression in children has been systematically evaluated through multiple meta-analyses. Sandali et al. reviewed AI prediction algorithms for refractive error onset and progression in children using non-image-based clinical data, reporting a pooled RMSE of 0.50D for spherical equivalent prediction across 16 studies, yet substantial methodological heterogeneity (*I*^2^ = 100%) limits the interpretability of this estimate (21). Wang et al. reported a pooled AUC of 0.85 for all models and 0.95 for optimal models at one-year follow-up for myopia onset prediction, with prospective designs outperforming retrospective ones (pooled AUCs of 0.96 and 0.89, respectively); however, only one of seven included studies was rated as low risk of bias using PROBAST + AI, and all were conducted in Chinese populations (22). Zuo et al. demonstrated an SROC of 0.98 for machine learning-based diagnosis and prediction of high myopia, with deep learning outperforming conventional machine learning (SROC values of 0.97 and 0.86 for pathological myopia, respectively), yet most included studies relied on retrospective data and the analysis domain of PROBAST flagged high risk of bias in 43.8% of models (23). Beyond structured clinical data, Yew et al. systematically reviewed deep learning models using retinal and external ocular photographs to predict refractive error, suggesting that imaging-based AI could serve as a large-scale screening tool (24). Across these meta-analyses and systematic reviews, consistent limitations emerge: substantial methodological heterogeneity, geographic concentration in East Asian populations, and insufficient external validation. These findings suggest that AI can stratify pediatric populations by myopia risk at a general level, yet the translation of predictive modeling to specific treatment contexts, such as predicting individual responses to orthokeratology, remains an emerging field with distinct challenges including variable baseline ocular parameters and heterogeneous treatment outcomes.

### 1.5 Aim, scope, and structure of this review

This review provides a systematic assessment of artificial intelligence applications across the full cycle of orthokeratology care, from initial patient screening to long-term safety monitoring. It aims to survey the current landscape of AI tools for prediction, fitting, and monitoring.

The analysis is structured in two parts: first, to detail the specific tasks AI is being developed for at each management stage, and second, to discuss the key hurdles preventing widespread clinical implementation. These hurdles include the scarcity of high-quality, diverse data for training models; the limited generalizability of models across different patient populations and clinical settings; and the practical challenges of integrating these tools into existing clinical workflows. Addressing these challenges will be essential for translating AI innovations into sustainable, real-world solutions for chronic ocular disease management (25).

This review synthesizes peer-reviewed literature on AI applications in orthokeratology, drawing on studies identified through searches of PubMed, Web of Science, and Scopus up to July 2026, supplemented by manual screening of reference lists. Priority was given to original studies reporting AI model development, validation, or clinical implementation at any stage of OK management, with emphasis on 2020–2026 literature to capture recent advances.

## 2 Core AI applications in full-cycle OK lens management

### 2.1 Pre-treatment: patient selection and efficacy prediction

#### 2.1.1 Predicting poor/good responders based on baseline data

Tang et al. developed a machine learning-based nomogram integrating baseline ocular parameters, including axial length and corneal curvature, to stratify patients by predicted treatment response (26). In a multicenter retrospective study of 1,588 Chinese children, the model achieved AUCs of 0.871 in training, 0.863 in internal validation, and 0.817 in external validation. While this represents one of the few externally validated models in the OK literature, its generalizability beyond Chinese children and Vision Shaping Treatment (VST) lens designs remains unconfirmed.

Meta-analytic evidence confirms that baseline refractive error predicts response magnitude in combination therapy. Zhang et al. found that adding atropine to orthokeratology produced significantly greater benefit in mild myopia than in moderate myopia, with the effect diminishing over longer follow-up periods. Most included trials were conducted in East Asian populations, and several did not fully report randomization methods (27).

The predictive value of baseline ocular biometry may be mediated by its interaction with corneal reshaping efficacy. Chong et al. demonstrated that the axial length to corneal curvature radius ratio (AL/CR) moderates the association between treatment zone corneal reshaping, quantified as summed relative corneal refractive power (sRCRP), and age-adjusted axial elongation. In 318 children followed for one year, sRCRP significantly predicted reduced axial elongation only in eyes with AL/CR below 0.61, a threshold that identifies eyes with relatively shorter axial length and steeper corneas (28). Above this ratio, the relationship between corneal reshaping and growth suppression weakened, suggesting that AL/CR may influence the optical-to-structural transduction of myopic defocus signals. The retrospective design and single-center sample limit generalization, and whether this moderating effect operates through optical or biomechanical pathways remains unresolved.

#### 2.1.2 Personalized prediction of myopia control efficacy

Cao et al. advanced this approach by developing a deep learning model to predict 12-month axial elongation continuously, achieving a test set R^2^ of 0.353 and RMSE of 0.115 mm (29). The shift from binary classification to quantitative prediction is conceptually valuable, yet the small sample (*n* = 115) and lack of external validation limit its clinical applicability.

Hu et al. conducted a three-year retrospective cohort study of 1,303 children wearing OK lenses, stratifying participants by baseline age (≤11 years and ≥11 years) and SE (≤-3.00D and ≥-3.00D) (30). Children with older age and higher myopia showed the best AL control (85.95% excellent response at year one) and the highest rate of AL regression (23.90% at two years). Those with younger age and lower myopia had the poorest control (13.21% excellent response at three years), suggesting that baseline demographic and refractive characteristics may influence the therapeutic ceiling of OK treatment. Wang et al. developed machine learning models incorporating first-year AL change as a dynamic predictor for long-term OK efficacy (31). In a retrospective cohort of 896 children, the model using baseline parameters alone achieved an AUC of 0.77 for first-year prediction, while the second-year model incorporating first-year AL change achieved an AUC of 0.95. However, the study was limited by a single-center design and the lack of external validation.

The same research group extended this evidence to longer follow-up periods. Wang et al. developed a five-year prediction model in a multicenter cohort of 504 children (32). First-year AL change (Δ1), third-year AL change (Δ3), and baseline flat eccentricity emerged as the most stable predictors, with logistic regression achieving an AUC of 0.969 in internal validation. SHAP analysis revealed a nonlinear threshold effect for Δ3 at 0.05 mm. Above this value, the predicted risk of poor five-year control increased sharply, suggesting that AL stabilization by the third year may signal sustained treatment efficacy. Extending the horizon to adulthood, Wang et al. conducted a ten-year longitudinal study of 101 children and found that first-year AL change, combined with baseline AL and flat eccentricity, predicted 10-year AL exceeding 26 mm with an AUC of 0.952 (33). An optimal first-year AL change cutoff of 0.28 mm was identified for identifying children at risk of poor ultra-long-term outcomes. However, both studies are limited by retrospective designs, and the near-ceiling AUC values suggest potential overfitting. The ten-year cohort was further constrained by a small sample size and approximately 30% attrition, raising concerns about selection bias. Prospective, multi-center validation is required before these models can inform clinical practice.

These studies suggest that baseline ocular parameters can serve as predictive biomarkers for identifying poor responders to OK treatment, allowing clinicians to adjust expectations and consider adjunctive therapies when appropriate. However, the transition from retrospective model development to prospective clinical deployment remains incomplete. Most OK prediction models lack validation beyond East Asian populations, and evidence for improved long-term myopia control outcomes compared with conventional patient selection remains limited. Multi-center validation across diverse populations and prospective studies demonstrating clinical utility beyond technical accuracy remain important priorities.

### 2.2 During treatment: precision and efficient fitting

#### 2.2.1 Intelligent prediction and recommendation of lens parameters

The conventional process for determining orthokeratology lens parameters is inherently iterative and experience-dependent. Clinicians select trial lenses based on examination data and refine choices through a cycle of patient adaptation and subjective feedback. This time-consuming process introduces significant variability, with inappropriate parameter selection compromising both safety and efficacy (34). Artificial intelligence offers a paradigm shift by predicting optimal lens parameters directly from baseline ocular data, potentially standardizing and streamlining the fitting process.

Studies from the early single-brand success of Chan et al. to the randomized trial by Sun et al. established that computer-aided fitting achieves comparable myopia control while significantly improving fitting efficiency and ensuring stable vision (35, 36).

Subsequent models have sought greater sophistication and generalizability. Zhou et al. developed a machine learning algorithm that demonstrated significantly smaller prescription deviations than traditional methods (37), while Yang et al. utilized a deep neural network to predict parameters, noting benefits such as reduced cross-infection risk (38). Both studies acknowledged limitations in handling extreme cases, highlighting the need for broader validation.

The field is advancing toward more robust and clinically integrated solutions. Koo et al. developed an expert-level model that predicts all major Corneal Refractive Therapy (CRT) lens parameters, overcoming the limitation of prior partial-prediction models (39). In a significant step toward generalizability, Xu et al. introduced a hybrid deep learning/machine learning model that standardizes inputs from different corneal topographers by solving key biomechanical relationships (40).

Zhang et al. developed a multimodal, multitask AI model that simultaneously recommends lens parameters and predicts axial elongation (41). Using 3,529 eyes fitted with VST lenses, their two-stage framework, a ResNet50-based recall model for parameter prediction and a CLIP-ViT-based ranking model for topography classification and AL growth prediction, achieved topography classification accuracies of 0.95–0.96 and AL growth prediction accuracies of 0.880–0.883. The model outputs multiple parameter candidates ranked by probability of achieving both optimal centration and slow axial growth (<0.3 mm/year). However, the model was developed exclusively for VST lenses and validated on single-center data, leaving its applicability to CRT designs and diverse populations unconfirmed.

Despite these promising developments, several challenges remain. Many existing models are trained on retrospective data from specific ethnic populations and lens designs, limiting generalizability. While studies like Fan et al. rigorously compare multiple machine learning algorithms, the ultimate clinical utility depends on validation through large-scale, prospective multi-center trials (42).

In conclusion, AI-driven parameter prediction has evolved into a clinically useful tool that can improve fitting accuracy, standardize care quality, and enhance accessibility via telemedicine. The shift toward clinically relevant metrics like first-fit success rates indicates growing maturity. Future progress requires multi-center validation across diverse populations and better integration with long-term myopia management.

Collectively, these studies mark a clear transition from proof-of-concept toward clinical implementation. Early research established the feasibility of computer-aided fitting (35, 36), followed by more sophisticated and accurate algorithms (37, 38, 42). Recent advances have improved generalizability across topographers and comprehensive parameter prediction (39, 40). However, most models remain limited by retrospective data and homogeneous training populations. Future work must prioritize prospective validation in diverse clinical settings to enable routine use.

#### 2.2.2 Automated assessment of lens fit

Beyond predicting initial lens parameters, AI is revolutionizing the post-fitting evaluation by automating the assessment of the corneal treatment zone (TZ)—a critical indicator of orthokeratology efficacy defined by its centration and boundaries. The accurate demarcation of this area is essential for evaluating treatment outcomes but is traditionally subjective and time-consuming.

Pioneering this domain, Tang et al. developed a deep learning framework based on Fully Convolutional Networks (FCNs) to automatically identify the TZ boundary and center from axial and tangential curvature difference maps (43). Their study, leveraging a substantial dataset of 6,328 images, demonstrated the fundamental feasibility of automating this task. The algorithm achieved promising results, offering a potential tool to streamline clinical workflow. However, the reported mean center deviation of 0.22 mm, particularly with their CNN-based center detection, underscores a critical limitation in precision for clinical applications where sub-millimeter accuracy is paramount. This early work effectively highlights the trade-off between automation and precision, establishing a benchmark for future models to surpass.

Shifting the focus from pure image analysis to clinical workflow efficiency, Yi et al. investigated a sagittal height simulation software (44). In a randomized controlled study on Chinese myopic children, they demonstrated that the software-calculated alignment curve (AC) radius could achieve a comparable fit to the traditional method operated by optometrists, but with a statistically significant reduction in the number of trial lenses required. Nevertheless, its scope was limited to the AC parameter, and its generalizability beyond the specific population and lens designs studied remains unverified.

Building upon the foundational work of Tang et al., Zhang et al. introduced a more advanced deep learning model with a self-attention mechanism to segment not only the TZ but also the peripheral steepened zone (PSZ) (43, 45). Their model achieved remarkable performance, with Intersection over Union (IoU) scores exceeding 94% for both zones (45). A particularly noteworthy finding was the model's high agreement with human experts in TZ segmentation, validating its clinical relevance. However, the significant discrepancy observed in PSZ segmentation reveals a greater complexity in defining this region and suggests that the model's performance may be susceptible to unseen topographic variations. This admission underscores that high IoU scores in a controlled test set do not automatically equate to robust clinical readiness, necessitating external validation.

Departing from the paradigm of replicating human assessment, Lin et al. asked a more clinically direct question: can AI predict the functional consequence of lens decentration (46)? Instead of merely measuring TZ displacement, they developed a model to predict the resulting magnitude and direction of induced higher-order aberrations (HOAs) and astigmatism. Their approach, which achieved a prediction accuracy of approximately 70%–72% for decentration direction, represents a conceptual shift from “evaluating the fit” to “predicting the patient's visual outcome”. While the 70% accuracy is a compelling starting point, it is not yet reliable enough for standalone clinical decision-making. This pioneering work points toward a future where AI could preemptively flag patients at risk of poor visual quality due to lens decentration, moving from passive assessment to proactive management.

Beyond structural assessment, the functional optical consequences of corneal reshaping have also been characterized. Wang et al. prospectively evaluated regional changes in relative peripheral refraction (RPR) after OK in 41 children using a refraction topographer, with single-vision spectacle wearers as controls (47). Relative peripheral myopic defocus increased significantly in all regions between 15° and 53° of the visual field except the nasal quadrant, with more pronounced changes temporally, inferiorly, and towards the periphery. The magnitude of RPR change correlated positively with baseline myopia severity and negatively with the prominence of the high convex zone, confirming that greater corneal reshaping generates stronger myopic defocus signals. However, the 2-month follow-up was too short to determine whether early RPR changes predict long-term myopia control efficacy.

The evolution across these five studies traces a clear path from automation of a manual task (43), to process optimization (44), to technical refinement (45), to outcome prediction (46), and finally to functional optical assessment (47). Yet, all five studies share a common limitation: they were trained and validated on narrow datasets from single centers or specific populations. Their performance in multi-center, real-world settings with diverse ethnic groups and different topographers remains untested. Whether the reported metrics, such as a 0.2 mm center deviation or 70% prediction accuracy, translate into meaningful gains in long-term myopia control or patient satisfaction has not been established. Without prospective, multi-center validation and direct evidence linking these AI-driven assessments to improved clinical outcomes, their practical value remains uncertain.

### 2.3 Post-treatment: safety monitoring and complication alert

#### 2.3.1 Tracking and analysis of corneal topography changes

Continuous monitoring of corneal morphological changes is essential for maintaining the long-term safety and efficacy of orthokeratology (48). Regular follow-up assessments track treatment zone characteristics, corneal integrity, and potential complications, forming the basis of clinical management through precise evaluation of OK-induced structural changes.

Sánchez-García et al. systematically reviewed corneal structural changes under OK therapy. Their analysis of 12 studies confirmed that OK lenses induce primarily reversible alterations in the anterior cornea (48). However, the review's limited scope, with most follow-ups lasting one year or less, constrains its ability to assess long-term safety. The authors acknowledge this evidence gap and call for more longitudinal studies.

Li et al. developed a machine learning model to address the clinical challenge of managing OK patients lacking historical fitting data (49). To solve this “missing parameter” problem, they developed a novel machine learning approach to predict the original, pre-treatment corneal curvature (K1 and K2) from post-OK corneal topography. Their study, utilizing data from 497 right eyes fitted with Paragon CRT lenses, demonstrated that Bagging Tree algorithms could achieve high predictive accuracy (R > 0.81). This approach streamlines clinical workflow by reducing the need for complete refitting, offering clear time and cost savings.

However, Li et al.'s model faces notable limitations (49). Its validation on a single lens brand (CRT) and specific population (Chinese children) limits generalizability. Additionally, the model cannot predict corneal eccentricity (e-value), a critical parameter for lens selection. These constraints underscore the challenges in developing universally applicable AI tools in ophthalmology.

Together, these studies highlight two complementary facets of post-fitting care: the need for a fundamental understanding of long-term corneal structural responses and the development of intelligent tools to manage practical clinical dilemmas (48, 49). The former exposes a gap in long-term biological evidence, while the latter demonstrates a technological solution that is, for now, contextually limited. Future work should validate AI models across diverse populations while conducting long-term safety studies.

#### 2.3.2 Early identification and prediction of complication risks

Post-treatment management requires the early identification of serious complications.

Maseedupally et al. quantified how corneal toricity predisposes Ortho-K lenses to decentration, directly linking misalignment to induced optical aberrations (13). This work translates a clinical observation into a quantifiable risk factor, providing the precise patterns of decentration that AI systems must learn to detect automatically from topographic maps for early intervention.

Cope et al. established the incidence and devastating outcomes of Acanthamoeba keratitis among RGP wearers, linking risk to lens hygiene and water exposure (14). This highlights the reactive nature of traditional care. AI enables proactive risk stratification by analyzing follow-up scans to detect subtle corneal changes that may indicate infection risk (43, 45). Furthermore, computer vision techniques, proven powerful in ocular image analysis, could be developed to analyze lens surfaces for harmful deposits, enabling pre-emptive safeguards (24).

These studies provide a roadmap for AI in safety monitoring, shifting the goal from tracking complications to proactively predicting and preventing them.

### 2.4 Full-cycle: patient education and compliance management

Large language models (LLMs) present a promising avenue for automating and scaling patient education and consultation in orthokeratology, a critical component for ensuring adherence and minimizing complications. Recent systematic reviews have established the broad potential of large language models in ophthalmology, highlighting their capacity to process natural language queries, generate patient-friendly content, and support clinical decision-making across various subspecialties (50). A recent scoping review further synthesized the emerging role of LLMs in chronic ocular disease management, highlighting their potential to support patient communication, treatment adherence, and long-term follow-up across diverse clinical settings (51).

Huang et al. directly assessed the viability of LLMs, including GPT-4 and Chinese-specific models like Qwen-72B, in providing accurate responses to a standardized set of OK lens care questions (52). Their findings confirmed the fundamental competency of these models, with GPT-4 and Qwen-72B both demonstrating high accuracy. However, the study revealed a critical nuance: while accurate, GPT-4's responses had lower readability, potentially impairing patient comprehension, whereas Qwen-72B achieved higher overall satisfaction due to its conciseness. This underscores that model performance is multi-faceted, and optimal performance may require language or region-specific model selection. A key limitation acknowledged was the small cohort of evaluating ophthalmologists, highlighting the need for broader clinical validation.

Building upon the foundation of feasibility, Delsoz et al. investigated the practical application of LLMs in generating patient education materials (PEMs) for pediatric myopia (53). Their work addressed a known barrier in patient communication: the excessively high reading level of most existing PEMs. The study demonstrated that LLMs, particularly ChatGPT-4o, could effectively create accurate PEMs and, crucially, rewrite complex texts to meet the recommended 6th-grade reading level, thereby significantly enhancing accessibility. This capability for readability optimization is a direct and valuable clinical application. However, Delsoz et al. also identified a significant functional gap: despite being understandable, all LLM-generated PEMs lacked actionable steps for patients, limiting their practical utility (54). This indicates that current LLMs are more effective at information transmission than at generating detailed, personalized action plans. Furthermore, the dependency on sophisticated prompt engineering to achieve these results was evident, and the generalizability of findings may be limited by the English-only, US-centric focus of the study.

Collectively, these studies affirm the considerable potential of LLMs to augment patient education by providing scalable, accurate, and accessible information (52, 53). However, they also delineate clear boundaries for their current use. These findings align with broader challenges identified in the ophthalmic LLM literature, including concerns about response accuracy, readability, cultural adaptability, and the need for robust validation frameworks before clinical deployment (54). Effective deployment is contingent upon careful model and prompt selection, and outputs must be evaluated for more than just accuracy, encompassing readability, cultural appropriateness, and the inclusion of actionable guidance. Future work must focus on multi-lingual and multi-modal integration, robust clinical validation, and developing frameworks to bridge the actionability gap to fully realize the promise of LLMs in supporting patient-centered care.Key findings of the studies discussed above are summarized in Table 1.

**Table 1:** Summary of key AI studies in orthokeratology management.

Study	Task	Key findings	Limitations
Pre-treatment: prediction			
Tang et al., (26)	Poor responder prediction (1-year axial elongation ≥ 0.20 mm)	AUC: 0.871 (training), 0.863 (internal), 0.817 (external); multi-center (*n* = 1,588)	Retrospective; VST lenses only; single ethnicity
Zhang et al., (27)	Meta-analysis comparing AOK with OK (11 RCTs)	WMD in axial elongation: −0.16 mm (95% CI: −0.22 to −0.10, *p* < 0.001); greater benefit in mild myopia (WMD −0.15 mm, 95% CI −0.21 to −0.10) than in moderate myopia (WMD −0.00 mm, 95% CI −0.08 to 0.08)	Most trials East Asian; incomplete randomization reporting in several trials
Thakur et al., (11)	Systematic review of combination therapy options	OK with atropine: additional reduction in axial elongation of 0.09–0.29 mm over 12–24 months compared with OK alone; clinical framework for fast progressors proposed	Mechanisms underlying synergy unclear; long-term safety data scarce
Cao et al., (29)	Myopia control efficacy prediction (12-month axial length)	Test set: R^2^ = 0.353, MAE=0.088 mm, RMSE=0.115 mm; combined linear regression and deep neural network (DNN)	Small sample (*n* = 115); single-center; no external validation
Wang et al., (31)	Short-term and long-term OK efficacy prediction using baseline and dynamic AL changes	Validation set: LightGBM AUC 0.95 (Year 2); SVM F1 0.73 (Year 3), 0.75 (Year 4); *n* = 896	Single-center; retrospective; 23% attrition
Wang et al., (32)	Five-year prediction model for OK long-term control outcomes	Logistic regression AUC 0.969 for 5-year AL control; Δ1 SHAP 0.14, Δ3 SHAP 0.18; *n* = 504	Retrospective; overfitting risk; requires external validation
Wang et al., (33)	10-year longitudinal study of first-year AL change and adult AL outcomes	First-year AL change cutoff 0.28 mm predicted 10-year AL >26 mm; AUC improved from 0.726 (cutoff alone) to 0.952 (combined with baseline AL and flat E); *n* = 101	Single-center; small sample; 30% attrition; selection bias
During treatment: lens fitting			
Chan et al., (35)	Computer-aided fitting (feasibility)	First-fit success: 90% (centration); SER reduction: 89% at 1 month	Early feasibility study; single brand; no control group
Fan et al., (42)	Multi-algorithm comparison (alignment curve prediction)	AC1K1: R^2^ 0.91 (SVM), MAE 0.263 D; AC1K2: R^2^ 0.84, MAE 0.396 D (Gaussian process); AC2K1: R^2^ 0.73, MAE 0.507 D (Gaussian process); trial attempts: 1.18 (ML) compared with 1.86 (traditional) per eye (*P* < 0.0001); *n* = 1,271	Retrospective; single center; VST lenses only
Yang et al., (38)	Parameter prediction (AC/TP/LD)	AC: MAE 0.21 D, R^2^ 0.97; TP: MAE 0.19 D, R^2^ 0.95; LD: MAE 0.02 mm, R^2^ 0.91; *n* = 449	Poor performance in extreme cases (K > 45 D, myopia < 1.00 D, astigmatism > 2.00 D)
Sun et al., (36)	Parameter prediction (randomized controlled trial)	1-month first-fit success: 93.6% in the trial group, 84.0% in the control group (*P* = 0.03)	Single lens design; 16.9% withdrawal; no advantage in axial length control
Zhou et al., (37)	Parameter prediction (CRT lenses)	Correlation with final lens (R): BCR 0.958, RZD1 0.708, RZD2 0.773, LZA1 0.697, LZA2 0.654, TD 0.730; AI predicted RZD2 (0.773) and LZA1 (0.697) closer to final lens than manufacturer's calculator (0.701 and 0.488, respectively); *n* = 797	Retrospective; single topographer (Medmont); CRT lenses only
Xu et al., (40)	Parameter prediction (AC/CP)	AC: MAE 0.136 D, R^2^ = 0.973; CP: ACC 0.898, AUC 0.896 (hybrid model)	Single lens brand; single topographer; single ethnicity
Zhang et al., (41)	Multimodal multitask AI for lens fitting and outcome prediction (parameter recommendation, topography classification, AL growth prediction)	Topography classification accuracy 0.95–0.96 across three models; AL growth prediction accuracy 0.880–0.883; lens parameter prediction MSE 0.18 D; ±0.25 D recall rate 0.94; *n* = 3,529	Single-center; VST lenses only; uniform 0.3 mm/year AL threshold across ages
During treatment: automated assessment			
Tang et al., (43)	TZ segmentation (FCN)	IoU 0.90 ± 0.06; center deviation 0.22 ± 0.22 mm; 6,328 images	Sub-millimeter precision lacking; Pentacam only; no external validation
Zhang et al., (45)	TZ and PSZ segmentation (self-attention-based DNN)	TZ IoU 0.981, PSZ IoU 0.945; TZ segmentation showed high agreement with human experts; PSZ segmentation more variable than TZ; *n* = 1,346 maps	Single-center; PSZ segmentation more variable than TZ; Medmont only
Lin et al., (46)	Decentration effect prediction (higher-order aberrations)	Direction accuracy: 70.2% (right), 71.8% (left); decentration RMS: 0.31 mm (right), 0.25 mm (left); *n* = 249	Accuracy insufficient for independent clinical decision-making; retrospective
Full-cycle: patient education			
Huang et al., (52)	LLM-based OK care Q&A (Chinese)	GPT-4 readability: 3.79; Qwen-72B readability: 4.65, satisfaction: 4.91 (5-point Likert scale)	Small question set (*n* = 24); single language; small evaluator cohort
Delsoz et al., (53)	LLM-based PEM generation	ChatGPT-4o: SMOG reduced from 10.3 to 5.3; FKGL reduced from 9.7 to 5.8 (rewriting existing online PEMs)	No actionable guidance; English-only; US-centric materials

AC, alignment curve; AOK, atropine combined with orthokeratology; AUC, area under the receiver operating characteristic curve; BCR, base curve radius; CP, cylinder power; CRT, corneal refractive therapy; DNN, deep neural network; FCN, fully convolutional network; FKGL, Flesch–Kincaid grade level; IoU, intersection over union; LD, lens diameter; LLM, large language model; LZA1, horizontal landing zone angle; LZA2, vertical landing zone angle; MAE, mean absolute error; ML, machine learning; MSE, mean squared error; PEM, patient education material; PSZ, peripheral steepened zone; R^2^, coefficient of determination; RMSE, root mean square error; RZD1, horizontal return zone depth; RZD2, vertical return zone depth; SER, spherical equivalent refraction; SHAP, SHapley Additive exPlanations; SVM, support vector machine; TD, total diameter; TP, target power; TZ, treatment zone; VST, vision shaping treatment; WMD, weighted mean difference; Δ1, first-year axial length change; Δ3, third-year axial length change.

## 3 Discussion

### 3.1 Technical and clinical limitations

#### 3.1.1 Data quality and algorithmic bias

The development of robust AI models for orthokeratology is fundamentally constrained by challenges pertaining to data quality and the mitigation of algorithmic bias (55). These limitations threaten the generalizability and fairness of AI applications across the care cycle.

A primary concern is the scarcity and homogeneity of high-quality, annotated datasets. As highlighted by Zhang et al., while research activity in AI for myopia is growing, there remains a pronounced lack of comprehensive biometric analyses and sufficient studies on prognostic prediction (56). This indicates that existing datasets are likely inadequate for training models that can reliably support long-term intervention decisions, as they fail to capture the full spectrum of patient phenotypes and outcomes. This data paucity is compounded by a lack of standardization across studies, which hinders the aggregation of data from multiple sources to create more robust training sets.

Furthermore, the use of limited or non-representative data directly introduces algorithmic bias. Nguyen et al. emphasize this risk, noting that AI models trained on narrow demographic or clinical cohorts may not generalize well to more diverse real-world populations (55). This could lead to disparities in performance across different ethnicities, age groups, or geographic regions. The iterative nature of AI development compounds this risk; if biased models are deployed and their outputs are used to inform future data collection, it can create a feedback loop that perpetuates and amplifies existing inaccuracies and inequities.

Proposed technical solutions, such as data augmentation with generative adversarial networks (GANs) or transfer learning, aim to overcome data scarcity. Similarly, explainable AI (XAI) methods are being developed to enhance model transparency and help identify sources of bias. However, as Nguyen et al. caution, the efficacy of these solutions requires rigorous validation through large-scale, multi-center clinical trials, which are currently scarce in ophthalmology (55). Therefore, resolving the issues of data quality and bias is not merely a technical exercise but a prerequisite for the ethical and clinically trustworthy integration of AI into orthokeratology practice.

Beyond dataset composition, the transparency of reporting itself is essential for assessing algorithmic bias and determining whether a model suits a given clinical context. Chen et al. reviewed 37 prospective validation studies of ophthalmic AI models and found substantial variability in reporting: while most studies reported intended use and performance metrics, demographic information of training data was rarely disclosed, with only 5 of 27 unique models reporting race or ethnicity. Scope of use was also poorly defined, with 15 of 37 studies failing to specify primary users (57). These gaps hinder clinicians from judging whether a model applies to their patient population. For orthokeratology, this implies that many AI-assisted fitting tools may lack sufficient documentation for clinicians to assess their applicability to specific patient subgroups. Alderman et al. developed the STANDING Together consensus recommendations, a 29-item framework for transparent documentation and use of health datasets in AI development, covering data origin, sampling strategies, group composition, and evaluation of performance across at-risk groups (58). The authors emphasized that no dataset is free of limitations, and transparent communication of these limitations should be valued rather than viewed as deficiency. For OK-specific models, the framework offers actionable guidance: dataset curators should report demographic composition and known biases in data acquisition; data users should evaluate performance across relevant subgroups. However, adoption requires resource investment that may challenge small teams without institutional support. Across these studies, the persistent gap between what is reported and what is needed to assess algorithmic fairness has direct implications for OK practice, where treatment outcomes vary by ethnicity and age. Without transparent reporting and diverse validation, clinicians cannot determine whether an AI-assisted fitting tool generalizes to their patients. Addressing this requires coordinated efforts from researchers, journals, and regulators to mandate adherence to reporting standards and ensure OK-specific AI models are accompanied by sufficient documentation for safe, equitable use.

#### 3.1.2 Model interpretability and clinical trust

The opacity of many complex AI models presents a significant barrier to their clinical adoption in orthokeratology. When model recommendations lack transparent reasoning, clinicians justifiably hesitate to trust and integrate them into high-stakes decisions, such as predicting long-term myopia control efficacy or identifying patients at risk for complications.

Zhang et al. indirectly underscore this critical gap through their bibliometric analysis (56). The notable scarcity of studies focused on prognostic prediction within the AI-myopia research landscape implies that the field has not yet adequately addressed the challenge of building models that not only predict but also explain their outputs. This absence of explainability-focused research fundamentally limits the translational potential of AI from research benchmarks to trusted clinical tools. For AI to effectively guide intervention decisions, clinicians require insight into why a patient is predicted to be a poor responder, not just the prediction itself.

The inability to interrogate a model's decision-making process has direct consequences. Without feature importance analysis or a clear rationale, a clinician cannot reconcile an AI's output with their own expertise or a patient's unique clinical circumstances. This erodes trust and prevents meaningful human-AI collaboration. Furthermore, the lack of interpretability complicates the identification and correction of underlying model biases, as Zhang et al. discussed, creating a cycle of opacity and mistrust (56). Therefore, advancing the field requires a deliberate shift from merely developing predictive models to creating interpretable, clinically-reasoned systems that can foster, rather than demand, clinical trust.

#### 3.1.3 Insufficient clinical translation and external validation

Many studies report high predictive accuracy in retrospective analyses but lack prospective multi-center validation. This gap is not confined to parameter prediction. In automated fit assessment, Zhang et al. achieved TZ segmentation IoU >94% on a single-center dataset, yet their model's performance on data from different topographers or ethnic groups remains untested (45). Similarly, Lin et al. reported 70% accuracy in predicting decentration direction, a threshold insufficient for standalone clinical decision-making (46). These examples illustrate that technical benchmarks do not equate to clinical readiness.

Among parameter prediction studies, Sun et al. and Xu et al. serve as further illustrations (36, 40). The former achieved >90% prediction accuracy in a multi-center trial but used a single lens design; the latter outperformed experienced practitioners in lens selection yet relied on data from one ethnic group and one topographer type. Both remain proof-of-concept for broader populations.

Bridging this gap requires prospective, multi-center trials that assess not only predictive accuracy but also workflow efficiency and patient outcomes. Without such evidence, AI's research performance cannot be translated into trusted clinical practice.

### 3.2 Ethical, privacy, and regulatory considerations

The integration of artificial intelligence into orthokeratology practice introduces complex ethical and regulatory challenges. On one hand, algorithmic bias poses an ethical risk, as unrepresentative training data can worsen healthcare disparities and threaten equitable care (55). On the other hand, the field must develop clear ethical guidelines and respond to regulations for AI-based medical devices. Together, these steps are essential to ensure the technology is implemented in a safe, effective, and fair manner.

Beyond legal and governance frameworks, the interpretability of AI models themselves constitutes a foundational ethical prerequisite for clinical adoption. When clinicians cannot understand why a model recommends a specific lens parameter or flags a complication risk, meaningful oversight becomes impossible, and the ethical obligations of informed consent and accountability remain unfulfilled.

An et al. systematically distinguished between explainable AI (xAI), which describes the holistic functioning of an AI system, and interpretable AI (iAI), which focuses specifically on understanding the algorithm's internal decision-making processes (59). Their review of iAI techniques applied to oculomics models revealed that current methods, particularly class activation maps and saliency maps, are limited to showing where a model looked in an image without clarifying what features it used or how they contributed to the prediction. For orthokeratology, where lens fitting decisions rely on subtle corneal topographic features, the inability to trace how an AI model arrived at a specific parameter recommendation directly undermines clinician trust and complicates accountability in cases of adverse outcomes. Teng et al. traced the evolution from conventional AI through explainable AI to trustworthy AI (TAI) in medical image segmentation, emphasizing that transparency alone is insufficient without robustness, safety, and accountability (60). Their analysis noted that *post-hoc* interpretability techniques can generate inconsistent explanations for the same prediction, implying that current XAI tools may not provide the reliable, reproducible explanations required for clinical decision-making in OK practice.

Kalaw and Baxter examined LLM applications in ophthalmology through the lens of the four foundational principles of medical ethics: beneficence, nonmaleficence, autonomy, and justice (61). Their review identified significant concerns including potential misinformation, breaches of patient privacy, and biases inherent in training datasets. For orthokeratology, these concerns translate directly into clinical risk: an AI model that confidently generates incorrect parameter suggestions could lead to suboptimal outcomes, yet current systems lack mechanisms to flag such uncertainty. Veritti et al. identified six core issues associated with AI use in ophthalmology, emphasizing that bias often arises from distributional shift, where models trained on homogeneous datasets fail to generalize to target populations (62). For OK lens fitting, this implies that models trained predominantly on East Asian populations may not perform equivalently for patients of other ethnicities, given known differences in corneal topography and myopia progression patterns. Beyond these ethical considerations, data security vulnerabilities and fragmented regulatory databases that lack AI-specific nomenclature further complicate the safe deployment of OK AI tools across jurisdictions (63, 64).

These insights carry direct implications for OK practice. First, the development of OK-specific AI models should prioritize intrinsic interpretability where feasible, for example through multi-staged architectures that break down complex predictions into clinically meaningful sub-tasks. Second, models must undergo rigorous external validation across diverse populations before clinical deployment. Third, the limitations of *post-hoc* visualizations should be explicitly communicated to clinicians, who must recognize that heatmaps indicate correlation rather than causation and may not reliably reflect model decision-making. Fourth, data security and regulatory transparency require coordinated efforts from developers, clinicians, and regulators to establish secure data governance and standardized reporting. Without deliberate attention to these dimensions, even technically accurate models risk being underutilized or misapplied, limiting their potential to improve myopia management outcomes.

### 3.3 Future directions

#### 3.3.1 Constructing multi-center diverse databases to mitigate data bias

The journey toward robust and equitable AI begins with its foundation: data. Future initiatives must prioritize the construction of large-scale, multi-center databases that capture a diverse spectrum of patient phenotypes through standardized protocols (55, 56). This requires establishing common data standards for imaging and outcomes to enable the creation of a powerful, shared resource. The approach has been highlighted as critical for advancing AI applications in orthokeratology (OK) fitting and myopia management (40). The ultimate goal is to develop AI that performs reliably for all patient groups, ensuring the technology benefits everyone equitably, not just the populations on which it was originally trained.

Specifically, such multi-center databases should encompass patients with varied demographic backgrounds (ethnicities, age groups, geographic regions) and corneal morphological characteristics (normal curvatures, extreme toricity, keratoconus tendency, irregular topography) (26, 55). Long-term OK treatment outcomes, including axial length changes, corneal reshaping dynamics, and complication records, must be integrated to strengthen the generalizability of AI models (26, 29). Multi-center retrospective studies have demonstrated that diverse phenotypic data enhances the predictive performance of machine learning models, underscoring the value of standardized data aggregation (26). Most existing OK-related AI models are trained on homogeneous datasets from specific ethnic populations, however, limiting their applicability across diverse real-world cohorts (40, 55).

Notably, systematic searches across major academic databases confirm a critical research gap. Targeted studies on constructing OK-specific multi-center databases for AI training remain scarce (56). While existing ophthalmic AI literature emphasizes the general importance of data diversity in mitigating algorithmic bias (55, 65), no research has translated this principle into practical frameworks for OK-specific multi-center data integration. Standardized data exchange protocols tailored to OK-related biometric data (e.g., corneal topography) across different clinical settings are additionally lacking. This is despite evidence that standardized corneal topography data improves AI model generalizability (40). Olawade et al. further highlight that algorithmic bias from homogeneous training data can lead to disparate AI performance across patient subgroups, reinforcing the urgency of addressing this gap (65).

To fill these gaps, database construction should incorporate three key measures. First, unified data collection protocols must be established across participating centers, adopting consistent standards for imaging acquisition and clinical data recording (40, 56). Second, bias detection mechanisms should be embedded, such as explicit labeling of demographic and phenotypic characteristics to identify underrepresented groups. Reweighting or resampling techniques can balance data distribution (55, 65). Third, adherence to international data standards, such as Health Level Seven Fast Healthcare Interoperability Resources (HL7 FHIR), is essential for interoperability between clinical systems (40). Data privacy must be safeguarded through robust encryption and anonymization (55).

The establishment of diverse, standardized multi-center databases will lay a solid foundation for equitable AI applications in OK practice. Future efforts should prioritize multi-center collaborations and unified data protocols to address the current research gap, ensuring AI benefits all patient populations equitably (26, 56). This aligns with the broader goal of reducing healthcare disparities in ophthalmic AI (65).

#### 3.3.2 Developing interpretable and generalizable AI models

With a solid data foundation in place, the next imperative is to build models that earn clinical trust and function reliably in the real world. The transition of AI from a research tool to a trusted clinical partner in orthokeratology hinges on overcoming the critical challenges of model opacity and limited generalizability (55, 56). As Zhang et al. observe, the absence of a comprehensive, full-cycle intelligent diagnosis and treatment system underscores a fundamental gap (56). When AI and human experts propose conflicting management plans, clinicians require more than just a predictive output. They need transparent insight into the AI's reasoning process to assess its credibility and inform clinical decisions.

This requires a dual focus: turning unexplainable systems into transparent partners and ensuring models are built to last. The integration of Explainable AI (XAI) is crucial to provide clinicians with insight into the ‘why’ behind a recommendation, transforming AI from an oracle into a consultable colleague (66). Technologies such as feature importance analysis, heatmap visualization, and model decomposition methods have been successfully used to clarify AI decision-making in retinal disease diagnosis. These tools enable clinicians to understand the rationale behind algorithmic outputs (66). For orthokeratology, XAI can map the relationship between corneal topographic features (e.g., central curvature, eccentricity) and AI-predicted lens parameters, or identify key indicators driving complication risk alerts (40, 45). Existing XAI research primarily focuses on fundus imaging, however. Adapting these techniques to corneal data, with its unique morphological characteristics and OK-specific clinical endpoints, remains an unaddressed research gap (66).

Concurrently, models must be stress-tested for generalizability across diverse clinical environments and robustness against imperfect, real-world data (55). The lack of OK lens AI models validated through multi-center studies means their performance across different ethnicities, corneal phenotypes, and imaging devices remains unproven (56). Ophthalmic AI research has demonstrated that models trained on single-center data often fail to maintain accuracy when deployed elsewhere. This is due to variations in data acquisition protocols and population characteristics (66). Strategies proven effective in enhancing generalizability include multi-center data integration, cross-device calibration, and the use of domain adaptation techniques to mitigate data distribution shifts (66). For example, entropy normalization and abnormal value exposure technologies have been shown to improve model robustness to non-target signals and data noise, with AUC values increasing by 5%–10% in retinal disease screening (67). These methods can be adapted to orthokeratology scenarios, such as handling topographic image artifacts or variations in corneal morphological features across age groups (40, 49).

The goal is to shift from merely predictive algorithms to trustworthy clinical reasoning systems that clinicians can understand and rely upon (55). Future efforts should prioritize adapting XAI technologies to corneal data characteristics and validating models across multi-center, diverse cohorts. By addressing these challenges, AI can transition from a research novelty to a trusted clinical tool seamlessly integrated into orthokeratology practice. This aligns with the broader goal of advancing reliable and equitable AI applications in ophthalmology (65, 66).

#### 3.3.3 Establishing a three-level clinical translation and validation system

The clinical value of AI technologies in orthokeratology can only be confirmed through systematic translation and multi-dimensional validation. This addresses the current lack of standardized evaluation frameworks for AI tools in this field (48). A three-level clinical translation and validation system encompasses technical performance verification, clinical utility assessment, and real-world effectiveness confirmation. It provides a structured pathway to bridge the gap between research and routine practice (50, 51). This hierarchical approach aligns with ophthalmic AI validation principles. It ensures that models are not only technically robust but also clinically relevant and adaptable to diverse care settings (46).

The first level focuses on technical performance verification, the foundational step for AI translation. Core metrics include accuracy, precision, recall, and AUC values for predictive models. They also include consistency and efficiency for automated analysis tools such as corneal topography segmentation (35, 39). Validation should leverage standardized, high-quality datasets. These datasets cover diverse corneal phenotypes, ethnicities, and imaging devices to ensure technical stability across variable inputs (18, 35). For example, AI models predicting orthokeratology lens parameters must demonstrate low prediction error across different age groups and corneal curvature ranges (68). Current technical validation often relies on single-center data. This limits the generalizability of performance metrics (48). Ophthalmic AI research highlights that technical excellence alone does not guarantee clinical value. This underscores the need for subsequent validation stages (50).

The second level centers on clinical utility assessment. It evaluates whether AI tools integrate effectively into clinical workflows and improve decision-making. Key indicators include reductions in fitting time, rate of lens parameter adjustments, and clinician cognitive burden (10, 51). This stage requires prospective clinical studies. AI tools are deployed in real consultation settings, with clinicians providing feedback on usability and relevance (52). For instance, an AI-assisted treatment zone evaluation tool should streamline follow-up assessments without compromising diagnostic accuracy (37, 69). Stakeholder research notes that clinical acceptance hinges on tools addressing specific workflow pain points. It does not depend on merely achieving technical benchmarks (52). A critical gap exists in orthokeratology-specific clinical utility studies. Most ophthalmic AI validation focuses on diagnostic tools for retinal or corneal diseases (50).

The third level involves real-world effectiveness confirmation. It is the ultimate test of AI's long-term value in diverse clinical environments. This stage requires multi-center, large-scale observational studies. They assess outcomes such as myopia control efficacy, complication rates, and patient satisfaction across different healthcare settings (18, 48). Validation should account for real-world variables. These include variable data quality, clinician experience levels, and patient compliance patterns (46). For example, entropy normalization and abnormal value exposure technologies improve model robustness to data noise in retinal screening (51). They can be adapted to enhance AI performance in real-world orthokeratology scenarios. Few AI models in orthokeratology have undergone such rigorous real-world validation. This reflects a critical barrier to widespread adoption (48).

Establishing this three-level system requires multi-stakeholder collaboration between AI developers, ophthalmic clinicians, regulatory bodies, and health informaticians (46, 52). Priority should be given to developing orthokeratology-specific validation guidelines. These include standardized datasets, core outcome measures, and reporting frameworks (35, 48). Regulatory involvement is essential to align validation requirements with safety and efficacy standards. This is evident in the approval process for ophthalmic AI diagnostic tools (52). Additionally, training programs should equip clinicians with the skills to participate in AI validation and interpret results. This addresses potential resistance stemming from unfamiliarity with technical metrics (10, 52).

This three-level clinical translation and validation system provides a rigorous, structured approach to advancing AI in orthokeratology (48). By systematically verifying technical performance, clinical utility, and real-world effectiveness, it mitigates risks associated with premature deployment. It also builds trust among clinicians, patients, and regulators (46, 52). Ultimately, this system accelerates the responsible translation of AI innovations into clinical practice. It ensures that technologies deliver tangible improvements in myopia control outcomes and care quality (49, 50).

#### 3.3.4 Building an AI-driven full-cycle management platform

The ultimate clinical value of interpretable and generalizable AI models, as well as effective human-AI collaboration, will be fully realized only when integrated into a unified platform that seamlessly embeds these capabilities into the complete orthokeratology workflow (48). Such a platform represents a paradigm shift from fragmented AI applications to holistic patient care, addressing the current disconnect between pre-treatment prediction, intra-treatment fitting, post-treatment monitoring, and patient education (46, 48). As highlighted in ophthalmic AI research, the true potential of technology lies not in isolated tools but in the synergistic capabilities created by their deep integration (50).

The core architecture of this AI-driven platform should center on three key pillars. First, it must enable continuous data interoperability across all clinical stages. Standardized corneal topography data (35), baseline ocular parameters (18), real-time lens fit assessments (39), and long-term treatment outcomes (19) should be aggregated into a unified patient record. This eliminates data silos that currently hinder comprehensive AI analysis, as demonstrated by the success of standardized data integration in other ophthalmic AI systems (50). Second, the platform should embed modular AI functions tailored to each management phase: predictive models for pre-treatment patient stratification (18, 19), intelligent parameter recommendation for lens fitting (35, 68), automated treatment zone evaluation for intra-treatment monitoring (37, 39), and risk alert systems for post-treatment complication prevention (43). Third, it should integrate patient education tools powered by large language models (44, 45), delivering personalized, accessible guidance that enhances adherence to lens care and follow-up protocols—addressing patient concerns about treatment transparency highlighted in stakeholder research (70).

Despite its theoretical promise, the development of such a platform faces practical challenges rooted in current research limitations. No existing studies have specifically designed or validated an AI-integrated full-cycle platform for orthokeratology, reflecting a critical gap in translating individual AI applications into cohesive clinical solutions (48). Ophthalmic AI integration efforts in other domains, such as diabetic retinopathy screening (14, 15), have encountered barriers including incompatible clinical workflows, variable data quality across centers, and resistance to technology-driven practice changes (50, 52). These lessons underscore the need for platform development to be clinically led, ensuring alignment with real-world optometric workflows rather than forcing clinicians to adapt to technological constraints (10, 46). Tailored approaches that account for diverse global healthcare contexts and stakeholder attitudes are also essential for successful adoption (52).

Future progress requires targeted multi-disciplinary collaboration between AI developers, ophthalmic clinicians, and health informaticians. Priority should be given to establishing universal data standards for orthokeratology-specific variables (35, 48), ensuring compatibility between different imaging devices and electronic health record systems. Additionally, platform validation must extend beyond technical performance metrics to include clinical utility assessments, such as improvements in fitting efficiency, reduction in complication rates, and enhanced patient satisfaction (51). Pilot studies in multi-center settings would provide critical evidence on scalability and adaptability across diverse clinical environments (46, 50). Addressing training gaps and liability concerns identified in stakeholder research will further facilitate platform integration into routine practice (52).

An AI-driven full-cycle management platform has the potential to redefine orthokeratology practice by standardizing care, improving accessibility, and shifting toward proactive, personalized myopia management (48). By addressing integration challenges, centering clinical relevance in platform design, and accounting for stakeholder perspectives (40, 52), AI can transcend its current role as a collection of isolated tools to become the backbone of a more efficient, equitable, and high-quality orthokeratology care system.

#### 3.3.5 Optimizing human-AI collaboration in clinical workflows

The successful integration of AI into orthokeratology practice ultimately depends not on technological sophistication alone, but on the quality of collaboration between clinicians and AI systems. Even the most accurate predictive models will fail to improve patient outcomes if they are not trusted, understood, or effectively utilized by the clinicians who care for patients.

A balanced framework for human-AI collaboration should define clear role boundaries. AI is best positioned as a decision-support tool that processes complex data and offers evidence-based recommendations, while clinicians retain ultimate responsibility for diagnosis, treatment decisions, and patient communication (12, 55). This division of labor leverages the computational strengths of AI without compromising the irreplaceable human elements of clinical practice, including contextual judgment, empathy, and ethical reasoning.

Several practical considerations are essential for fostering effective collaboration. First, user interface design must prioritize transparency and interpretability, allowing clinicians to quickly understand the rationale behind AI recommendations (66). Second, training programs should equip eye care professionals with the skills to critically evaluate AI outputs, recognize their limitations, and determine when to override algorithmic suggestions (70). Third, clinical workflows need to be redesigned to accommodate AI tools seamlessly, ensuring that they augment rather than disrupt routine practice (10, 52).

Addressing these human factors is as critical as improving model performance. Without deliberate attention to how clinicians and AI systems interact, even the most advanced technologies risk being underutilized or misapplied, ultimately limiting their potential to improve myopia management outcomes.

Beyond clinician-AI interaction, systemic barriers further complicate clinical integration. Chen and Bai (2025) reviewed AI applications in ophthalmic public health and identified interoperability with electronic health records as a primary obstacle (72). Existing standards such as HL7 FHIR, while effective for general medical data, lack the metadata standardization required for ophthalmic imaging modalities including OCT and fundus photography. For orthokeratology, this means corneal topography data from different devices may not conform to unified formats, complicating both real-time fitting assistance and longitudinal treatment monitoring. The authors also noted that AI systems often degrade in performance over time, yet the frequency and cost of recalibration remain largely unaddressed. Chong et al. examined AI integration across multiple domains and found that most models remain at the proof-of-concept stage, constrained by limited generalizability from non-representative datasets and substantial computational demands that restrict deployment in resource-limited settings (71). For OK practice, technical accuracy in retrospective studies does not guarantee clinical utility. Evidence of benefit beyond current practice remains sparse, and few studies have addressed validation across diverse settings or workflow integration. Prospective implementation studies are needed.

#### 3.3.6 Incorporating multimodal AI and foundation models

Recent advances in multimodal AI and foundation models represent a conceptual shift in how AI systems are developed and deployed in ophthalmology. Unlike task-specific models trained on narrow, labeled datasets for single applications, foundation models are pretrained on large-scale, heterogeneous data and can be adapted to multiple downstream tasks with minimal labeled data. This paradigm offers particular relevance to orthokeratology, where clinical decisions rely on multiple data types, including corneal topography, axial length measurements, and refraction history, yet these are currently interpreted in isolation rather than through integrated AI-driven analysis.

Jin et al. conducted a systematic review of vision and vision-language foundation models in ophthalmology, demonstrating that these models achieved diagnostic accuracy comparable to or exceeding experienced clinicians across retinal diseases, glaucoma, and ocular surface tumors, with key advantages including cross-task transferability and multimodal input processing (73). However, their review also identified persistent limitations: algorithmic bias from training data concentrated in specific populations, substantial computational requirements, and opaque decision-making processes. Notably, the reviewed studies focused exclusively on retinal diseases, glaucoma, and ocular surface tumors, with no identified applications in orthokeratology or myopia control interventions.

Shen et al. examined the application of multimodal AI to automated report generation in ophthalmology, integrating fundus photography, optical coherence tomography, and clinical data through deep learning and natural language processing (74). While representative systems demonstrated improvements in diagnostic accuracy and reporting efficiency, most remain at the proof-of-concept stage with limited prospective validation.

Hao et al. proposed a broader framework for multimodal integration across health care, outlining three interconnected barriers: data standardization, model interpretability, and deployment infrastructure (75). Their analysis noted that combining multimodal imaging and clinical data achieved high diagnostic accuracy in glaucoma detection, yet model generalizability remained constrained by reliance on specific devices and patient populations.

Across these reviews, certain themes warrant consideration for orthokeratology. While foundation models have not yet been applied to OK practice, their architectural advantages, including cross-task transferability and multimodal integration, suggest potential for combining the diverse data types encountered in OK care. The challenges identified in these reviews, including data heterogeneity, limited interpretability, and insufficient external validation, mirror those documented in the OK-specific AI literature discussed in earlier sections, reinforcing that these barriers must be addressed systematically across the field. Future work may explore the feasibility of OK-specific foundation models and validate their performance in real-world clinical settings.

### 3.4 Conclusion

The efficacy and safety of modern orthokeratology for myopia control in children have been firmly established through clinical evidence (5). The integration of artificial intelligence represents a significant advancement that promises to enhance therapeutic outcomes (5, 12, 56). Not all AI applications, however, are equally ready for clinical use.

AI-assisted lens parameter prediction has the strongest evidence. Multi-center randomized trials and externally validated models show that AI algorithms determine initial lens parameters with accuracy comparable to or exceeding experienced practitioners, reducing fitting time and trial lens requirements (36, 39, 40). AI-assisted treatment zone evaluation is technically mature for clinical use, though evidence linking these improvements to long-term myopia control outcomes remains limited (43, 45).

Pre-treatment efficacy prediction, by contrast, remains investigational. Models developed by Cao et al. and Tang et al. require prospective multi-center validation across diverse populations before clinical deployment. Multimodal foundation models and vision-language systems are at the proof-of-concept stage, with no OK-specific validation to date (26, 29).

These gaps point to clear priorities. Large-scale, multi-center, ethnically diverse databases with standardized protocols are needed to address current generalizability limitations. Prospective validation of existing prediction models through multi-center trials is required to establish real-world performance. Regulatory frameworks must also evolve to accommodate adaptive AI algorithms.

For clinicians, AI-assisted lens fitting tools can be adopted with appropriate oversight, but outputs should be verified against clinical judgment. Predictive models for treatment response should inform rather than dictate decisions. For researchers, the priority is prospective validation and clinical utility assessment.

Ultimately, the greatest potential lies not in autonomous AI systems but in synergistic human-AI collaboration, where AI augments rather than replaces clinical judgment (12, 34).

### 3.5 Limitations of this review

Several limitations of this review should be acknowledged. The review may be subject to publication bias, as studies reporting positive or statistically significant results are more likely to be published. Language restrictions may also have excluded relevant evidence published in other languages, and the search was restricted to three databases (PubMed, Web of Science, and Scopus). The rapid pace of AI development means that even with emphasis on 2025–2026 publications, the review may not capture the most recent advances, especially in large language models and foundation models.

The predominance of studies conducted in East Asian populations limits the generalizability of findings to other geographic regions and ethnic groups. The narrative synthesis approach, chosen due to substantial heterogeneity in study designs, outcome definitions, and performance metrics, does not permit quantitative pooling of results. Finally, this review is dependent on the completeness and transparency of reporting in primary studies, and inadequate reporting in some included studies may have constrained the depth of analysis.
